# The Impact of the Environment on Pediatric Patients During Computed Tomography Exams: Experience from a Tertiary Center

**DOI:** 10.3390/diagnostics15192448

**Published:** 2025-09-25

**Authors:** Sonia Triggiani, Giuseppe Pellegrino, Simone Mortellaro, Alessandra Bubba, Chiara Grilli, Giorgia Schiraldi, Mauro Borrella, Veronica Magni, Francesco Iandola, Andrea Coppola, Giulia Pacella, Maria Chiara Brunese, Maria Giovanna Riga, Sveva Mortellaro, Massimo Venturini, Gianpaolo Carrafiello

**Affiliations:** 1 Post-Graduation School of Diagnostic and Interventional Radiology, University of Milan, 20122 Milan, Italy; sonia.triggiani@unimi.it (S.T.); giuseppe.pellegrino@unimi.it (G.P.); simone.mortellaro@unimi.it (S.M.); alessandra.bubba@unimi.it (A.B.); chiara.grilli@unimi.it (C.G.); giorgia.schiraldi@unimi.it (G.S.); mauro.borrella@unimi.it (M.B.); veronica.magni@unimi.it (V.M.); sveva.mortellaro@unimi.it (S.M.); 2 Fondazione G. e D. De Marchi ETS, 20122 Milan, Italy; info@fondazionedemarchi.it; 3 Diagnostic and Interventional Radiology Unit, Circolo Hospital, ASST Sette Laghi, 21100 Varese, Italy; coppola.andrea.mg@gmail.com (A.C.); massimo.venturini@uninsubria.it (M.V.); 4 Department of Medicine and Technological Innovation, Insubria University, 21100 Varese, Italy; 5 Department of Medicine and Health Science “V. Tiberio”, University of Molise, 86100 Campobasso, Italy; mariachiarabrunese@gmail.com; 6 Department of Medicine DIMED, University of Padua, 35123 Padua, Italy; mariagiovannariga@gmail.com; 7 Department of Health Services, IRCCS Ca’ Granda Fondazione Ospedale Maggiore Policlinico, 20122 Milan, Italy; gianpaolo.carrafiello@unimi.it; 8 Department of Oncoematology, University of Milan, 20122 Milan, Italy

**Keywords:** computed tomography (CT), environment, healthcare, humanization of care (HOC), pediatric

## Abstract

**Aim:** To assess the impact of the environment on the attitude and mood of pediatric patients undergoing computed tomography (CT) scans at our pediatric clinic within the colorful setting of a dedicated CT scan room. **Background:** In pediatric healthcare, interventions must address the specific needs of both children and their parents. While medical imaging is essential for diagnosis and management, it can cause stress and anxiety in children, potentially affecting cooperation and diagnostic quality. Creating a supportive, child-friendly environment can alleviate these challenges and improve the likelihood of successful imaging outcomes. **Method:** This retrospective quantitative study investigated the phenomenological experience of pediatric patients (aged 0–18) who underwent CT imaging at the “De Marchi” pediatric clinic between November 2021 and June 2024. Patients were manually selected from the clinic’s CT database. A standardized telephone survey assessed perceived environmental impact before, during, and after the procedure. Data were anonymized and recorded in Excel (Microsoft, Redmond, WA, USA). Crosstab analyses and Pearson’s chi-square tests were performed using SPSS version 25.0 (IBM, Armonk, NY, USA). **Results:** Between November 2021 and June 2024, 838 pediatric patients underwent 1144 CT scans at the “De Marchi” clinic. Patients were grouped as previous-CT elsewhere (12.3%), first-time CT (68.9%), and returning patients (18.8%). Most reported positive experiences: 63.3% of the first group rated the experience better than prior exams, 73.9% of first-time patients found the environment comfortable, and 85.6% of returning patients felt at ease. Overall, 94.2% would recommend the clinic for pediatric CT scans. **Conclusions:** Our research demonstrates that a child-friendly environment is not just a concept, but a tangible solution that effectively alleviates the stress experienced by young patients and their families during diagnostic exams. This finding should inspire optimism about the potential for creating more reassuring and comfortable hospital settings in pediatric healthcare.

## 1. Introduction

### 1.1. Background

**Figure 1:**
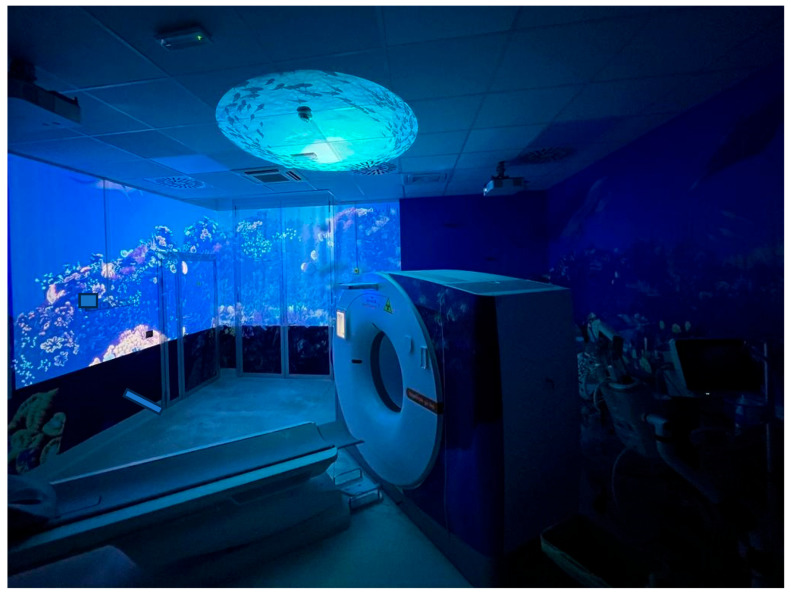
CT room of the pediatric radiology department of “Fondazione IRCCS Ca’ Granda Ospedale Maggiore Policlinico” of Milan.

The concept of humanization of care (HOC) is gaining increasing importance in healthcare, especially within the framework of patient-centered medicine [1]. HOC refers to an approach that respects the individual’s dignity and holistic needs [2], addressing not only physical health but also emotional, psychological, social, cultural, and environmental factors. In pediatrics, this approach is particularly critical due to the unique vulnerability of children and their families. Children are not simply “small adults”—they experience illness, hospitalization, and medical procedures in distinct, emotionally charged ways [3]. Despite its recognized value, implementing HOC in pediatric settings can be challenging. Healthcare systems often prioritize efficiency, advanced technology, and clinical outcomes, sometimes overlooking the emotional and psychological needs of young patients. Clinical and radiological environments can be intimidating, but child-friendly settings—incorporating appropriate colors, imagery, lighting, and distractions—along with effective communication with parents can reduce distress and improve cooperation [4,5]. By integrating technical excellence with emotional support, HOC promotes safer and more compassionate pediatric care, particularly during procedures such as contrast-enhanced CT scans, where anxiety may otherwise compromise outcomes [6,7,8]. Emergency departments often worsen this distress [9] due to noise, overstimulation, and the lack of pediatric-specific design [10]. While some children are treated in pediatric EDs, most receive care in general settings not tailored to their needs [11,12]. According to supportive design theory, reducing environmental stressors and enhancing coping mechanisms through thoughtful design can improve outcomes; in fact, studies confirm that children prefer pediatric environments, which contribute to a sense of safety and comfort [13]. In this context, the “Pedrito” project was launched in 2021 at the Pediatric Radiology Department of Fondazione IRCCS Ca’ Granda Ospedale Maggiore Policlinico in Milan. Sponsored by the De Marchi Foundation, the project aimed to transform the CT room into a child-friendly environment, reducing anxiety and improving the diagnostic experience for young patients (Figure 1).

### 1.2. Objectives

This study aims to evaluate the impact of the environment within the CT room on the attitudes and moods of pediatric patients undergoing examinations in our pediatric radiology department. Specifically, we aim to investigate how children benefit from the colorful design of the dedicated CT scan room in terms of their confidence, feelings of safety, and potential future examinations. Additionally, we seek to propose recommendations for implementing humanized care practices within the pediatric radiology department.

## 2. Materials and Methods

### 2.1. Study Design and Setting

This retrospective study evaluated pediatric patients’ experience in the newly designed CT room (Somatom go.Top, Siemens Healthineers, Erlangen, Germany) at the pediatric radiology department of “De Marchi” Clinic, IRCCS Ca’ Granda Ospedale Maggiore Policlinico, Milan.

The room features a calming, underwater-themed design that uses 3D projections, colorful murals, and soothing sounds to create a child-friendly environment. The immersive atmosphere alleviates anxiety and stress in pediatric patients during CT scans.

### 2.2. Patient Population

The study retrospectively included all outpatients and inpatients aged 0–18 years who underwent a CT scan between November 2021, when the new CT room was implemented, and June 2024. Using our machine databases, we identified the patients and subsequently attempted to contact them by telephone. The contacts were often made through the parents of the minor patients. Of the 1000 patients contacted, 838 (83.8%) completed the questionnaire, while the remaining individuals either did not respond or lacked a recorded mobile phone number in the electronic register.

### 2.3. Questionnaire and Data Collection

A telephone interview was conducted to assess the impact of the environment before, during, and after the diagnostic exam. After obtaining informed consent, radiology residents administered the satisfaction questionnaire during the call. The satisfaction questionnaire, developed by experienced radiologists from our foundation, was designed to be accessible and easily understandable for both patients and their parents. Specifically, the questionnaire avoids medical terminology and aims to evaluate the CT examination experience in relation to the environment, focusing on whether it contributed to the comfort of the experience. The questionnaire, created specifically for this study, addressed multiple aspects of the patient’s interaction with the CT room and the procedure, with an emphasis on emotional and physical comfort (Supplement S1). The term “comfortable” refers to an environment perceived as calm, reassuring, and welcoming, allowing patients to feel at ease. Finally, the questionnaire was validated and reviewed by the office and the privacy officer of our hospital. Responses were recorded digitally.

### 2.4. Statistical Analysis

Data were collected from questionnaires completed directly by radiology residents during telephone interviews, utilizing online forms that automatically recorded the provided responses. Data were anonymized and collected in an electronic dataset (Excel, Microsoft, Redmond, WA, USA). For descriptive statistics, numbers and percentages were presented, since we had only categorical variables. Crosstabs and Pearson’s chi-square test were used to assess relations among variables; to reduce the risk of type I error due to multiple comparisons, *p*-values were adjusted using the Bonferroni correction. In addition to *p*-values, effect sizes were calculated using Cramer’s V to evaluate the strength of associations, with values interpreted as weak (0.1–0.3), moderate (0.3–0.5), or strong (≥0.5). SPSS version 25.0 (IBM, Armonk, NY, USA) was used for all statistical analyses. *p*-values were considered significant when they were <0.05. Data visualization was achieved through the use of pie charts and histograms.

## 3. Results

**Chart 1:**
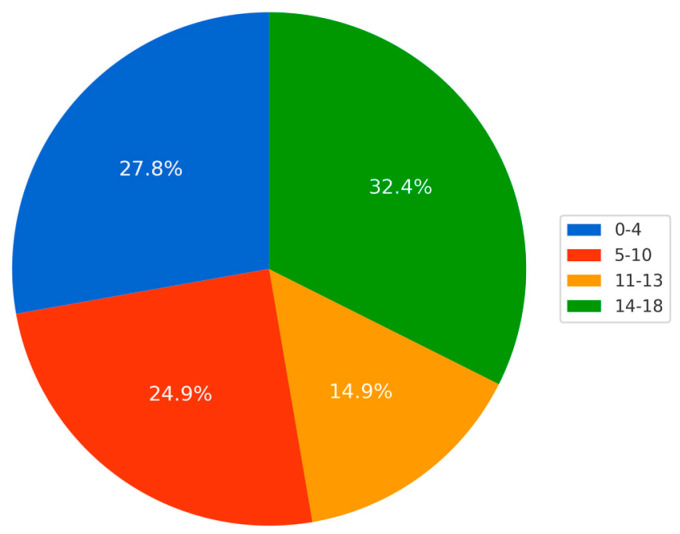
Distribution of the age of children referred to “De Marchi” Clinic for CT and that filled in the questionnaire.

In 92.4% of cases, the parents of the young patients answered the questionnaire. In 6% of cases, the patients responded, and in 1.7% of cases, patients and family members answered together. All patients were under 18 years old at the time of the diagnostic examination: 32.4% were aged 14–18 years, 27.8% were aged 0–4 years, 24.9% were aged 5–10 years, and 14.9% were aged 11–13 years (see Chart 1 and Table 1). Regarding gender distribution, 56.1% of the patients were male, and 43.9% were female. The hospital pediatrician prescribed 94.3% of CT examinations, while only 5.7% were prescribed by a family pediatrician. This discrepancy can be attributed to several factors, such as managing congenital diseases from birth, including cystic fibrosis or urological conditions, for which the “De Marchi” Clinic is a referral center. Additionally, many cases involved emergency room visits, where the hospital pediatrician evaluated the patients. Consequently, most respondents––approximately 473 individuals (56.7%)––indicated that the hospital or a family pediatrician recommended the Clinic. In 41.5% of cases, the Radiology Unit was recommended for other reasons, such as proximity during emergency room visits or transfers from different hospitals. In comparison, only 15 patients (1.8%) reported that the recommendation came from friends or family (Table 1).

In total, 68.9% of the patients had never undergone a CT scan before the study. Among this group, the majority expressed positive feedback regarding the CT room environment: 37.3% strongly agreed and 36.6% agreed that the design and colors of the room enhanced their overall experience. Additionally, 24.3% were neutral (neither agreed nor disagreed), while only 1.7% disagreed, and none strongly disagreed. Of the patients, 18.8% had previously undergone CT imaging at this Clinic. Among these individuals, 48.1% agreed that the environment and colors of the CT room enhanced their experience, while 37.5% strongly agreed. Additionally, 14.4% neither agreed nor disagreed with this statement, and none disagreed or strongly disagreed. In total, 12.3% of the young patients had previously undergone CT imaging at other centers. Among these patients, 43.3% agreed that the CT environment at the “De Marchi” Clinic improved their experience compared to other hospitals, while 20% strongly agreed. Additionally, 30% neither agreed nor disagreed with this statement, 6.7% disagreed, and none strongly disagreed. A total of 94.2% of patients either strongly agreed (52.6%) or agreed (41.6%) that they would recommend the center to others. Additionally, 5% were neutral (neither agreed nor disagreed), 0.7% disagreed, and none strongly disagreed (Table 1).

Additional data regarding the correlations identified through the chi-square test are presented in Table 2.

**Chart 2:**
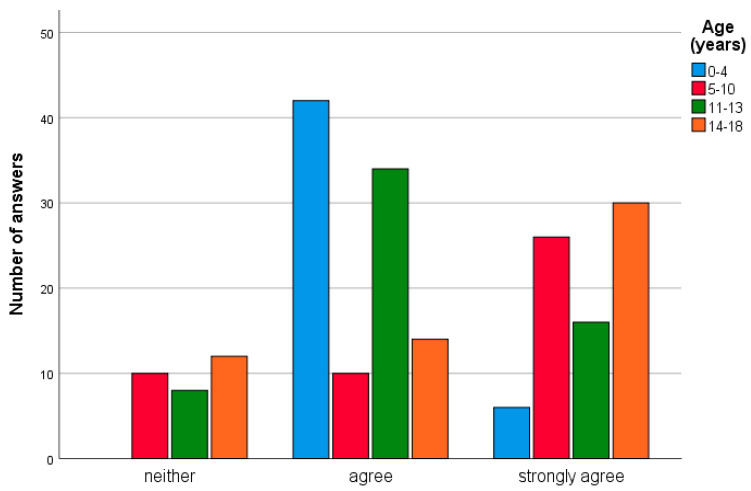
Histogram showing the different age groups that answered the question “If you routinely have CT follow-up or you have had a CT more than once in this Clinic, do you feel comfortable coming into this Clinic?”.

Pearson’s chi-square and Cramer’s V test showed a moderate statistically significant correlation between the “If you routinely have CT follow-up or you have had a CT more than once in this Clinic, do you feel comfortable to come in this Clinic?” question and the age group (χ^2^: 58.07; *p*-value < 0.001; V: 0.4): no patient showed discomfort in repeating examinations to our clinic. An interesting spike of agreement seems to be evident in the 0–4 years group (Table 2 and Chart 2).

**Chart 3:**
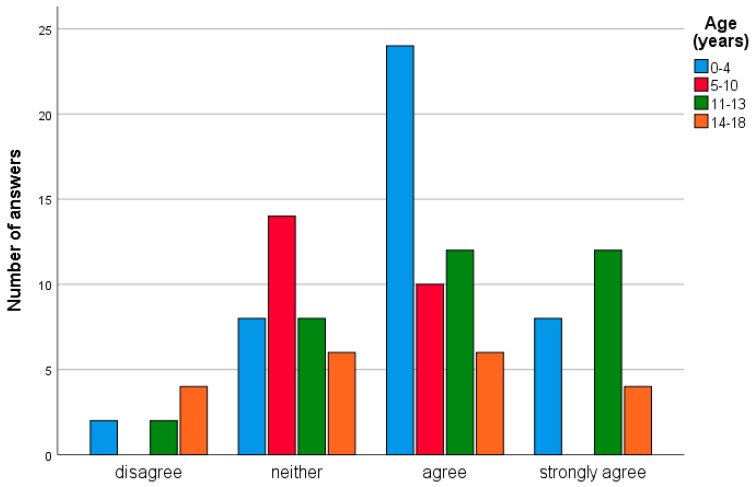
Histogram showing the different age groups that answered the question “If yes in another hospital, has the experience improved compared to other locations?”.

Moreover, a moderate statistically significant correlation was found between the “If yes in another hospital, has the experience improved compared to other locations?” question and the age group (χ^2^: 27.76; *p*-value = 0.013; V: 0.3). Again, the 0–4 years group seemed to be more interested (Table 2 and Chart 3).

A moderate statistically significant correlation between the “If yes in another hospital, has the experience improved compared to other locations?” question and the prescribing physician was also noted (χ^2^: 16.21; *p*-value = 0.012; V = 0.4). The patients sent by family pediatricians seemed to be more neutral.

**Chart 4:**
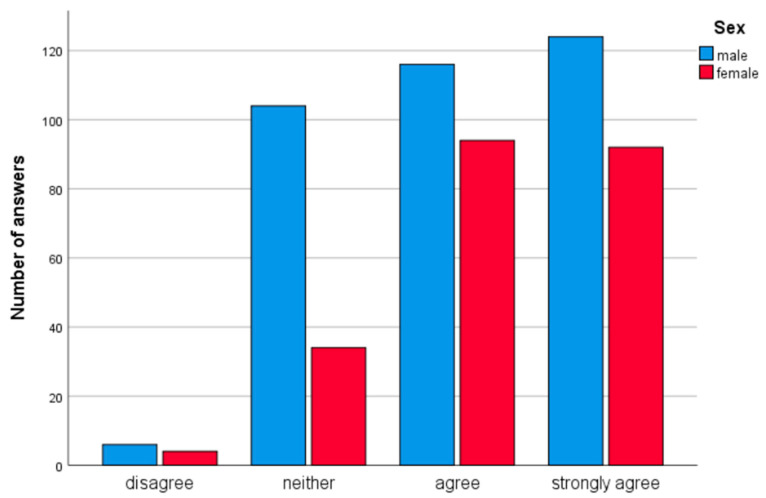
Histogram showing the different sex groups that answered the question “If not (this is your first time for a CT scan), did the environment and colors of this room make your experience more comfortable?”.

While for the previous questions there was no association with patient sex, for the question “If not (this is your first time for a CT scan), did the environment and colors of this room make your experience more comfortable?”, male patients seemed to be more interested in the environment than female patients ((χ^2^: 16.07; *p*-value = 0.013; V = 0.2) (Table 2 and Chart 4).

## 4. Discussion

### 4.1. Interpretation of Results

The results show how the environment and colorwork of the CT scan, in most cases, improve the experience, making children quieter during the exam and creating a more familiar place capable of reducing fear and suffering. These data reflect the experience of responders, both in an emergency context and for long-term follow-ups, who confirmed greater tranquility. In total, 788 responders would recommend the “De Marchi” Clinic. When analyzing the data, it is vital to consider the heterogeneous nature of the population, which ranged in age from a few months to 18 years. It is a hypothesis that some respondents who selected “neither agree nor disagree” did so because they may have been too young to fully comprehend the environment, or because the examination was performed under sedation, which may have limited their perception of the setting. Conversely, some older patients may have been less interested in or influenced by the CT room design. A small number of respondents expressed disagreement, often for reasons not directly related to the CT room itself—such as dissatisfaction with older hospital corridors or long waiting times in the ED. These external factors contributed to a less positive overall experience.

This study aimed to evaluate patient perceptions of comfort and experience in a pediatric CT imaging setting, with particular attention to how age, sex, prior imaging history, and referring physician influenced responses. Several statistically significant associations were observed, highlighting the role of demographic and contextual factors in shaping patient satisfaction and comfort.

The Pearson’s chi-square test demonstrated a significant correlation between age group and the question regarding comfort in returning to the clinic for routine or repeated CT examinations (χ^2^ = 58.07, *p*-value < 0.001). Importantly, no patient expressed discomfort with the prospect of undergoing repeated imaging at our institution, suggesting a generally positive perception of the environment and the care provided. Interestingly, the 0–4 years age group showed a notably high level of agreement, which may reflect either parental impressions for this age cohort or a particularly child-friendly atmosphere tailored to younger children [14]. This finding underscores the importance of age-appropriate environmental design in pediatric radiology. A further significant association was observed between age and the perception of whether the experience in our clinic was improved compared to other facilities (χ^2^ = 27.76, *p*-value = 0.013), with the 0–4 years group again showing the most favorable responses. These trends may point to the effectiveness of targeted environmental and procedural modifications aimed at easing anxiety in very young children, such as the use of colorful surroundings or the involvement of caregivers.

The same question—comparing experiences across facilities—also correlated significantly with the prescribing physician (χ^2^ = 16.21, *p*-value = 0.012). Patients referred by family pediatricians appeared to respond more neutrally, which might reflect differences in pre-examination counseling or expectations set by the referring physician. This suggests a potential area for enhancing interdisciplinary communication to ensure consistent messaging and patient preparation across referring sources.

Although no significant associations with patient sex were observed for most questions, a notable exception emerged regarding the influence of environmental factors on first-time patients. For those undergoing CT imaging for the first time, male respondents reported significantly greater comfort associated with the room’s colors and overall environment (χ^2^ = 16.07, *p*-value = 0.013). This result may indicate that male patients, or perhaps their parents, place higher value on environmental aesthetics and may be more responsive to sensory aspects of the imaging experience.

Collectively, these findings emphasize the multifactorial nature of patient satisfaction in pediatric imaging. Environmental design, prior experience, age-related needs, and referring pathways all play a role in shaping perceptions. Future efforts should aim to further personalize the imaging experience, particularly for younger children and first-time patients, by integrating patient-centered design and communication strategies. Additionally, closer collaboration with referring physicians may help ensure that expectations and information provided prior to imaging align with the patient experience offered at the clinic. These observations suggest that, while the CT room’s child-friendly design may positively influence patient experience, it is only one of several contributing elements. Other aspects of the hospital journey—such as staff interactions, wait times, and sedation practices—likely play significant roles as well. Therefore, although the overall feedback was generally positive, and the environment appears to support a more humanized approach to care, these findings should be interpreted with mindful consideration of their context. Each detail makes the experience less traumatic for children and their parents.

In the end, the overall experience of the respondents was generally positive. HOC has been shown to play a crucial role in both the child–parent relationship and the psychological well-being of young patients.

### 4.2. Comparison with Existing Literature

**Figure 2:**
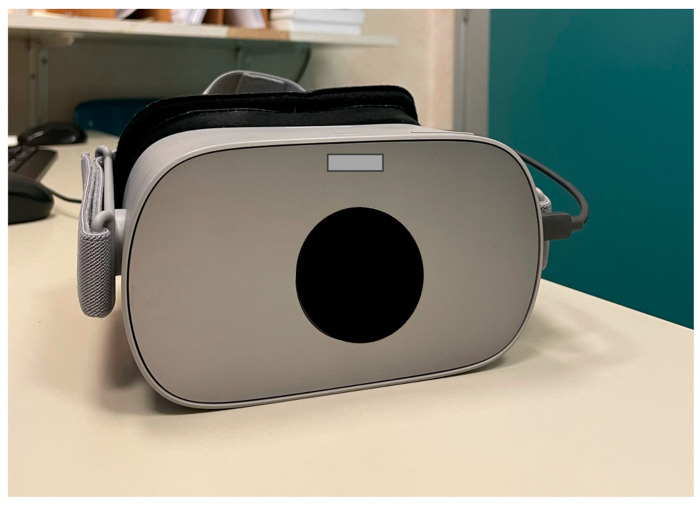
3D visor available for patients at the “De Marchi” Clinic.

Humanization of care (HOC) is a growing priority in healthcare, especially in pediatrics, where children’s unique emotional and developmental needs require a more holistic, patient-centered approach. Rather than viewing the child as a passive recipient of care, HOC emphasizes their active role, alongside that of their family, in the therapeutic process. This approach includes respect for their emotions, beliefs, and perception of illness [4,15]. Recent research has increasingly recognized the impact of physical environments on patients’ emotional states, stress levels, and overall health outcomes, particularly in children [16,17]. In pediatric care settings, sterile, unfamiliar, or intimidating environments can heighten fear and reduce cooperation. Conversely, welcoming, colorful, and interactive spaces have been shown to reduce anxiety and improve the overall experience. Pictorial interventions, in particular, have demonstrated benefits for both children and their families by creating emotionally supportive and less clinical atmospheres [18]. Despite this growing awareness, very few studies have specifically examined the influence of environmental design in diagnostic imaging settings, such as CT rooms, on pediatric patients. Our study addresses this gap by evaluating patient and caregiver feedback following the introduction of a child-friendly CT environment at the “De Marchi” Clinic in Milan, designed as part of the Pedrito Project in collaboration with the De Marchi Foundation. Our data show that environmental design plays a crucial role in shaping the pediatric patient experience: 94.2% of respondents stated they would recommend the facility, citing the welcoming environment as a key factor. Among first-time CT patients, 73.9% reported that the environment helped them feel more at ease. Of those who had previously undergone CT scans at other institutions, 43.3% agreed that the environment at De Marchi improved their experience, with 20% strongly agreeing. Among follow-up patients, 48.1% agreed and 37.5% strongly agreed that the environment had a positive influence on their return visits. The 0–4 age group showed the highest level of comfort and exam success, suggesting a strong effect of environmental factors on young children and their caregivers. These findings align with supportive design theory [13], which posits that environments that reduce stressors and provide positive distractions can improve emotional well-being and healthcare outcomes. A warm, visually engaging, and interactive setting helps children feel more in control and less threatened, which may reduce pre-procedure anxiety and encourage cooperation during exams. Moreover, our findings highlight the interconnected role of parents in the pediatric care experience. A well-designed space not only comforts the child, but also reduces parental anxiety, increases their perception of quality care, and strengthens their engagement in the clinical process. The literature suggests that design features such as ambient lighting, child-friendly decorations, and interactive tools contribute to a calmer and more reassuring atmosphere for both children and parents [19]. In line with this, the “De Marchi” CT room includes customizable elements like wallpaper selection and the use of 3D visors, offering age-appropriate distractions that empower the child and make the exam feel less intimidating. These personalized features not only improve comfort, but also foster a sense of control, which is particularly important for pediatric patients in unfamiliar medical settings. Adolescents, too, have shown a clear preference for pediatric-specific spaces over general hospital environments, which they often describe as overwhelming or intimidating [20,21]. Even when the decor is primarily designed for younger children, the supportive and comforting atmosphere appears to benefit older patients as well. Finally, beyond aesthetics, the functional aspects of design—such as adjustable lighting, temperature control, and quiet waiting areas—are essential. These features help accommodate diverse sensory needs, contributing to sensory-friendly environments that are increasingly important in inclusive pediatric care [22]. For this reason, a 3D visor is available in the “De Marchi” Clinic, and each patient can decide on the wallpaper with which to interact (Figure 2). In conclusion, our findings strongly support the notion that the physical environment in diagnostic imaging settings can influence pediatric patient outcomes and satisfaction. A thoughtfully designed, child-centered space not only improves the clinical experience, but also contributes to emotional well-being and fosters trust in the healthcare system. Future research should continue to explore the measurable impact of environmental factors on both clinical efficiency and patient-centered outcomes in pediatric radiology and beyond.

### 4.3. Implications for Practice

As previously shown in literature, environmental factors (such as bright colors, painting, and noises) have an essential impact on pediatric patients, making the hospitalization experience, including instrumental examination, less traumatic [23]. Our study demonstrates that a child-friendly environment during diagnostic procedures, specifically CT scans, can substantially reduce stress and anxiety in pediatric patients, thereby enhancing their comfort and cooperation throughout the examination. Importantly, the concept of a child-friendly environment is not limited to a specific theme, such as the “underwater” design used in our center; rather, it encompasses broader elements such as lighting, sounds, and other sensory stimuli that can be adapted to create a calming and engaging atmosphere. Our findings indicate that the physical environment plays a critical role in reassuring both patients and their parents [24], fostering greater confidence in the medical staff, consistent with previous literature [23]. The most important clinical implication of these findings is that, if the child is more comfortable, they tend to be more compliant during medical procedures. Regarding radiological exams, this mean that there is a greater probability that the patient will not move during the scan, improving the quality of the images and reducing the need for repeated scans, thereby avoiding exposure to additional radiation. Moreover, this prevents the need to sedate the patients during the exam. In this study, 260 patients underwent repeated CT scans, most for chronic conditions. An environment that makes the child feel comfortable during diagnostic procedures reduces fear of future procedures [25]. Additionally, if the parents feel comfortable and confident, they will be more likely to seek medical care at the same Clinic. The humanization of pediatric care presupposes intervention in different areas, like child-friendly environments, patient-medical relationships, technology, etc. [3]. This study emphasizes explicitly that a child-friendly environment, with colors, paintings, and soft and colorful light, can reduce the negative emotional impact on children and their families during diagnostic procedures. According to these findings, in radiological departments where children undergo examinations, the environments should be less austere, more comfortable, and more relaxing than in hospital wards. Even in resource-limited hospitals, it is possible to improve the impact of the physical healthcare environment with low-cost, high-impact interventions such as the creation of child-friendly murals or illustrations, by engaging volunteers, local artists, or students. For HOC, family-centered care and shared decision-making are very important in pediatric settings [23]. Furthermore, a holistic view of the patient is required, moving from evaluation of the disease itself to assessment of the disease in the context of the person [3]. According to these principles, it is necessary for the medical staff working in pediatric settings to be adequately trained to communicate with children and their families to make them feel safer and more confident. Even though the environment outside of the CT room was not included in the aim of this study, the literature conveys that the opportunity of having toys, books for children, and music available in the wards and waiting rooms can reduce the alienation of hospital settings and make children feel like they are in a familiar environment [4]. For this reason, creating a playful environment should become standard practice in hospital settings that children access. Based on our findings, renovating hospital facilities to create a more welcoming environment for children is essential for humanizing care. This approach aims to reduce disease-related stress in pediatric patients and contribute to diagnostic success. This approach potentially reduces the risks of adverse reactions, stress for the child, and consequently stress for the parents, who feel more reassured in a tailor-made environment, especially with younger children [19].

### 4.4. Study Limitations

This study has several limitations that may affect interpretation of its findings. As a retrospective analysis, it is subject to inherent biases related to data availability and the inability to control for confounding variables. The reliance on patient contact via mobile phone excluded individuals without accessible contact information, potentially introducing selection bias and limiting the generalizability of the results. Given the pediatric population, most questionnaires were completed by parents; responses were analyzed collectively to preserve sample representativeness, while acknowledging that parental input may reflect a proxy perspective. In some cases, patients were either too young or under sedation during their CT exam, making it difficult for them to recall or report sensations accurately. This may have led to underreporting of relevant subjective experiences.

### 4.5. Future Challenges

Future research should investigate the impact of environmental factors on pediatric patients in diverse clinical settings, such as inpatient wards, waiting areas, and ultrasound rooms. Moreover, prospective studies are needed to compare perception and experience across various pediatric age groups.

## 5. Conclusions

The limited attention given in the current literature to the role of the physical healthcare environment during pediatric diagnostic exams—such as CT scans—highlights the significance of our study. By focusing on environmental features such as child-friendly design, lighting, and colors, our findings contribute to a deeper understanding of how these elements can positively influence the pediatric patient experience. A child-friendly environment in CT rooms has been shown to alleviate discomfort in both pediatric patients and their parents, thereby enhancing safety awareness for future medical examinations. Evaluating the effects of reducing stress-related emotions on requirements for sedation and general anesthesia in pediatric care is essential. As highlighted by our findings, the environment is an integral component of humanizing pediatric care; hence, future studies could investigate the influence of environmental factors outside the computed CT room and within other pediatric radiology settings, including ultrasound, plain radiography, and magnetic resonance imaging. Even in settings with limited financial resources, meaningful improvements can be achieved through low-cost, high-impact strategies—such as the use of color, child-friendly artwork, improved lighting, and engaging signage. A multidimensional approach, involving community collaboration like engaging students or local artists, could facilitate private donations for the creation of libraries or play areas that address the specific needs of pediatric patients.

## Figures and Tables

**Table 1:** Percentage and numbers of responses given to the questionnaire questions, as well as age and gender distribution of the study population.

	Number	%
Who filled in the questionnaire?		
Parents	774	92.4
Patients with the help of family members	14	1.7
Patients	50	6
Age group		
0–4	232	27.8
5–10	208	24.9
11–13	124	14.9
14–18	270	32.4
Sex		
Male	462	56.1
Female	362	43.9
Who prescribed the visit and/or the exam?		
Family pediatrician	48	5.7
Hospital pediatrician	788	94.3
Who recommended this Clinic to you?		
Family/hospital pediatrician	473	56.7
Others	346	41.5
Friends/family members	15	1.8
Have you ever undergone a CT scan before or after this exam?		
No	572	68.9
Yes, in this clinic	156	18.8
Yes, in another hospital	102	12.3
If you routinely have CT follow-up or you have had a CT more than once in this Clinic, do you feel comfortable coming in to this Clinic?		
Strongly disagree	0	0
Disagree	0	0
Neither	30	14.4
Agree	100	48.1
Strongly agree	78	37.5
If yes in another hospital, has the experience improved compared to other locations?		
Strongly disagree	0	0
Disagree	8	6.7
Neither	36	30
Agree	52	43.3
Strongly agree	24	20
If not (this is your first time for a CT scan), did the environment and colors of this room make your experience more comfortable?		
Strongly disagree	0	0
Disagree	10	1.7
Neither	142	24.3
Agree	214	36.6
Strongly agree	218	37.3
Would you recommend this Clinic to others?		
Strongly disagree	0	0
Disagree	6	0.7
Neither	42	5
Agree	348	41.6
Strongly agree	440	52.6

**Table 2:** Results of chi-square (χ^2^) and Cramer’s V analyses conducted to evaluate the association between three demographic variables (age, sex, and prescribing physician) and questions regarding patients’ experiences at the Clinic.

	Age			Sex			Prescribing Physician		
	χ^2^	*p*-Value	Cramer’s V	χ^2^	*p*-Value	Cramer’s V	χ^2^	*p*-Value	Cramer’s V
If you routinely have CT follow-up or you have had a CT more than once in this Clinic, do you feel comfortable coming in to this Clinic?	58.066	<0.001	0.37	5.257	>0.05	0.17	17.676	0.0017	0.29
If yes in another hospital, has the experience improved compared to other locations?	27.756	0.013	0.28	4.991	>0.05	0.2	16.212	0.012	0.37
If not (this is your first time for a CT scan), did the environment and colors of this room make your experience more comfortable?	18.15	>0.05	0.1	16.068	0.013	0.17	5.404	>0.05	0.1
Would you recommend this Clinic to others?	23.37	>0.05	0.01	30.281	<0.001	0.19	46.28	<0.001	0.24

## Data Availability

The original contributions presented in this study are included in the article/Supplementary Material. Further inquiries can be directed to the corresponding author.

## Supplementary Materials

The following supporting information can be downloaded at: https://www.mdpi.com/article/10.3390/diagnostics15192448/s1.
