# Supplementary material for: The Impact of the Environment on Pediatric Patients During Computed Tomography Exams: Experience from a Tertiary Center
Source: Diagnostics (Basel). 2025 Sep 25;15(19):2448. doi: 10.3390/diagnostics15192448 (PMC12524014; doi:10.3390/diagnostics15192448)
Supplement: Supplementary file 1 [file diagnostics-15-02448-s001.zip › diagnostics-3811749-supplementary.pdf]

Supplement S1

	Question	*Answer*
1	Do you agree to the processing of your personal data, in accordance with EU Reg-ulation 2016/679 (GDPR) and Italian Legislative Decree 196/2003 as amended by Legislative Decree 101/2018, for scientific research purposes within the scope of this retrospective study, and to take part in the study?	Yes/No
2	Who fills in the questionnaire?	Patient/ Patient's parents/ Patient with the help of family members or medical staff
3	Age	0–4/ 5–10/ 11–13/ 14–18
4	Sex	Male/ Female
5	Who prescribed the exam?	Family pediatrician/ Hospital pediatrician
6	Who recommended this Clinic?	Family pediatrician/ Hospital pediatrician/ Friends/ Family members/ Others
7	Have you ever undergone a CT scan before or after this exam?	Yes, in this Clinic/ Yes, in another hospital/ No
8	If you have had multiple CT scans at this Clinic, do you feel comfortable coming here?	Strongly agree/ Agree / Neither / Disagree/ Strongly disagree
9	If you had a CT scan at another hospital, has the experience improved compared to other locations?	Strongly agree/ Agree / Neither / Disagree/ Strongly disagree
10	If this is your first CT scan, did the environment and colours of the room make your experience more comfortable?	Strongly agree/ Agree / Neither / Disagree/ Strongly disagree

11	Would you recommend this Clinic to others?	Strongly agree/ Agree / Neither / Disagree/ Strongly disagree
